# Internal Mechanisms of Human Motor Behaviour: A System-Theoretical Perspective

**DOI:** 10.3389/fpsyg.2022.841343

**Published:** 2022-04-28

**Authors:** Wacław Petryński, Robert Staszkiewicz, Mirosław Szyndera

**Affiliations:** ^1^Department of Tourism, Katowice Business University, Katowice, Poland; ^2^Department of Physical Education and Sport, University of Physical Education, Kraków, Poland; ^3^Department of Tourism and Recreation, University of Physical Education, Kraków, Poland

**Keywords:** human motor operation, modalities’ ladder, information processing in motor operation, mathematical description, system-theoretical description

## Abstract

The authors present the conceptual and system-theoretical model of human motor behaviour. The main assumption is that movement is the only observable manifestation of all psychical processes, thus, it is the only phenomenon enabling the creation of hypotheses concerning the psychological conditioning of human behaviour. They pointed to the fact that in the field of biology, and all the more, in psychology, mathematical descriptions are hardly eligible. In this respect, a system-theoretical approach seems to be appropriate. The authors present two systems: information processing modalities in the human mind, based on Nikolai Bernstein’s theory, and the series of processes from stimuli reception to motor response execution. Both these sub-systems make up a super-system. Its simplified graphical representation may be termed “Column Diagram.” The authors analyse the functioning of this super-system in various intellectual-motor purposeful operations. The system-theoretical perspective enables clear categorisation of various human motor operations, their “driving” mechanisms, internal patterns, and their superficial physical and/or mathematical “appearance.” The stream of consciousness in a human motor operation joins the various psychological constructs, which are reception, perception, attention, motivation, intellect, memory, etc., into one coherent, inseparable system.

## Introduction

Since the seventeenth century, when Isaac Newton published his seminal work “*Philosophiae naturalis principia mathematica”* (Newton, 2018), only physics, attired in a mathematical skirt, became the main engine of the whole of science. In 1814 Pierre Simon de Laplace invented the all-knowing Laplace’s Demon and wrote:

*We ought then to regard the present state of the universe as the effect of its anterior state and as the cause of the one which is to follow. Given for one instant an intelligence which could comprehend all the forces by which nature is animated and the respective situation of the beings who compose it—an intelligence sufficiently vast to submit these data to analysis—it would embrace in the same formula the movements of the greatest bodies of the universe and those of the lightest atom; for it, nothing would be uncertain and the future, as the past, would be present to its eyes* (de Laplace, 1902, p. 4).

The statement “*nothing would be uncertain*” sounds luscious, indeed, but nature is by far more complicated (and sometimes vicious). Nowadays it is clear to us that Laplace was too optimistic, and the “mathematical skeleton” of our world is not as hard as we wish it to be. The “mathematical engine” of science is very powerful, indeed, but it can drive only knowledge of a specific kind. It is highly effective in the non-living world, where the physical subjects passively obey the “stiff” laws external to them, which may be easily described mathematically. The items under investigation do not “actively” influence the relations joining them, and mathematical formalism makes very comfortable “rails for thinking,” which release the scientist from arduous reasoning, while a given problem has been already described mathematically. This is why some of Albert Einstein’s equations turned out to be “*wiser than Einstein himself”* (Coyne and Heller, 2008, p. 122).

However, the biological system not only passively obeys extrinsic physical laws but also actively shapes its relations with the environment. In biological organisms, and even structures, appear a new and important element: the intrinsic purposefulness, which actively influences the relations of the organism and the environment. Although the mechanisms of such shaping are relatively stable, developed during evolution, their mathematical description becomes hardly possible. This is why in the field of biology, the system approach has been invented by von Bertalanffy (1968).

The situation is still more complicated in psychology. In this case, the relations between an individual and the environment are shaped by at least three factors:

• Stiff physical laws, extrinsic to an individual;
• Somewhat elastic biological constraints developed for a given species during evolution; and quite fugacious psychological determinants, created uniquely by the individual.

Let us term the science on human motor behaviour “anthropokinetics.” According to Ann VanSant, it comprises motor control, which works in the period of milliseconds; motor learning—in hours, days, weeks; and motor development—in months, years, decades (VanSant, 2003).

Nevertheless, only well-ordered knowledge deserves the noble title “science.” In anthropokinetics, the promising perspective seems to be a system-theoretical approach (Petryński, 2016a). Table 1 has been shown the system of sciences, which describes and enables the understanding of the process of motor behaviour creation and execution in living beings (especially in humans).

**TABLE 1:** The system of sciences on motor behaviour of a human (Petryński, 2019, p. 29).

Task	”Actor”	Field	Sub-discipline	Discipline
Motor	Mind	Psychology		
operation			Anthropokinetics	
invention				
Motor operation	Nervous system	Neurophysiology		
control				Kinesiology
Motor operation	Musculoskeletal system	Physiology, anatomy		
execution			Biomechanics	
Operation results	Environment	Physics		

## The Instruments for Knowledge Ordering: Mathematics and System

In three centuries, B.C., Euclid already remarked, *“the laws of nature are but the mathematical thoughts of God*.” Carl Friedrich Gauss declared mathematics the “*Queen of Sciences.”* One of the most eminent mathematicians in history, David Hilbert, stated:

*Every kind of science, if it has only reached a certain degree of maturity, automatically becomes a part of mathematics.*

If this is true, then such a phenomenon marks a point, where the development of the “*kind of science*” starts to slow down. Because mathematics is not a fully universal instrument, enabling effective solving of every problem, but a science “from this point—to that point.” It is useful, or even extremely useful, in the non-living world, where the physical bodies passively obey the laws extrinsic to them. While establishing a network of such laws and describing them mathematically, it becomes possible to precisely anticipate the behaviour of such bodies. The outstanding mathematician and physicist, Nobel Prize winner Roger Penrose, stated:

*There are two other words I do not understand—*awareness *and* intelligence. *Well, why am I talking about things when I do not know what they mean? It is probably because I am a mathematician and mathematicians do not mind so much about that sort of thing. They do not need precise definitions of the things they are talking about, provided they can say something about the connections between them* (Penrose, 1997, p. 100).

Jim Holt quotes mathematician Alexander Grothendieck (Fields Medal laureate), who claimed that “*if you want to know the real nature of a mathematical object, don’t look inside it but see how it plays with its peers*” (Holt, 2018, p. 86). Hence, mathematics deals merely with relations, and not with the essence of items under consideration. As goes the popular joke, “*An engineer thinks that his equations are an approximation to reality; a physicist thinks reality is an approximation to his equations; a mathematician does not care.*” Accordingly, in the sciences regarding living creatures, the statement by Hilbert may be paraphrased in such a form:

*Every kind of science, if it only loses a hard ground of evident understandability and simple explainability under its feet* (e.g*., based on “new, original experimental data”*), *it automatically reaches for its lifebelt—mathematics.*

Unfortunately, such a lifebelt merely enables, in certain cases, passively drifting on the surface of knowledge, the understanding of which resides somewhere in the depth. Jack Cohen and Ian Stewart stated:

*Mathematics wallows in emergent phenomena. It also came to terms, long ago, with something that often puzzles non-mathematicians. By definition, mathematical statements are tautologies. Their conclusions are logical consequences of their hypotheses. The hypotheses already “contain” the information in the conclusions. The conclusions add nothing to what was implicitly known already. Mathematics tells you nothing new* (Cohen and Stewart, 1994, p. 234).

Therefore, Michał Heller wrote:

*For centuries we have worked out the empirical-mathematical method of world research. It is extremely efficacious, but for some price. It does not discern everything. Some things are transparent to it* (Heller, 2014, p. 295; transl. WP).

This statement includes, in fact, ominous content. The application of mathematics (or even sheer calculations) to issues where it is not eligible, may bring about disastrous results (O’Neil, 2016). Still earlier, in 1964, Garland Ashley published a paper entitled “*A declaration of independence from the statistical methods*” (Ashley, 1964). He remarked that statistics solves equations and not problems. In short, mathematics may produce models, whereas science (and practise, as well) needs, first of all, the interpretations. Summing up, it seems appropriate to quote the outstanding mathematician, Israel Moiseevich Gelfand, who also dealt with the biological issues:

*There is only one thing that is more unreasonable than the unreasonable effectiveness of mathematics in physics, and this is the unreasonable ineffectiveness of mathematics in biology* (Borovik, 2018).

This is no doubt a bon mot, and as such, it cannot be a source of scientific knowledge. However, it may contain a scientific truth. As, Hugo Steinhaus remarked, “*a joke, which is only a joke, is not a joke*” (Steinhaus, 1980, p. 47). So, it must include a certain idea, sometimes even a deep one. Gelfand was not only one of the most outstanding mathematicians of the 20th century, but also quite closely cooperated with Nikolai Bernstein. At their first meeting, when Bernstein presented his ideas, Gelfand murmured: “Rubbish… *rubbish*…*rubbish*.” But some years later, when he went along with Iosif Fejgenberg after Bernstein’s funeral through the snowbound Moscow, he stated: “*We have just buried a great mathematician.*” Without a doubt, Bernstein’s neurophysiological and evolutionary theory somehow influenced Gelfand’s mathematical mind. So, his aphorism (dubbed by Mark Latash “Wigner-Gelfand principle”) means that mathematics is not better than any other branch of science. It is highly, or even extremely efficient in some regions of knowledge, but not equally efficient beyond the kingdom of the Queen of Sciences. To apply it rationally in these “beyond regions,” a scientist must realise, how its limitations are. They may result from the fact that mathematics is too “stiff” for biology.

This rule might be termed the “dictatorship of the equals sign.” The same idea has been, slightly differently (and more concisely), expressed by Henri Poincaré, who stated that “*mathematics is the art of giving the same names to different things*.” However, Aristotle had already remarked, “*the whole is greater than the sum of its parts.”* Unfortunately, in a mathematical equation there is no place for any “greater.” On the contrary, a system is more elastic, and—first of all—it can produce a qualitatively new, unpredictable, emergent system effect (Morawski, 2005, p. 156; Petryński, 2016a, p. 6; Petryński, 2019, p. 24). As Lucien Cuénot stated, “*nothing is living in a cell but the whole cell*” (Gánti, 1986, p. 29; transl. WP). Therefore, in biology, the emergent system effect is life, and in psychology—the mind.

Incidentally, the term “emergent” means that—at least in motor control in humans—a system has a disposable structure. It consists of environment, body, mind, and task. After reaching its aim, it vanishes and leaves only a trace in memory termed “engram” (Semon, 1921). To solve next time a similar or even identical task, the performer has to build a new system. This makes a basis for what Bernstein termed “repetitions without repetitions.” It makes one of the fundamentals of his theory, and at the same time made “bone of contention” between him and his great predecessors—Ivan Mikhaylovich Sechenov and Ivan Petrovich Pavlov.

In short, the whole of science is being built of interpretations. Mathematics yields some solutions, which in the non-living world may be identified with interpretations. Nevertheless, in a living world, solutions merely make up a basis for systemic interpretations. Mathematics may yield a “bare” solution, which creates only space for reasoning and interpretations. Nothing more. This is why we suggest looking at human behaviour from a systemic perspective, not so user-friendly and unambiguous like mathematics, indeed, yet by far more elastic.

Hence, mathematics can be used only for the superficial description of biological or psychological phenomena and processes, but it hardly contributes to their understanding. This may be exemplified with the “evidence-based assessment,” which does not include any understanding; in fact, it vividly resembles the infamous behaviouristic “black box.” It is devoid of understanding, which makes it a vital component of any theory. However, the science is being “woven” just of the theories. Accordingly, mathematics is not a “Queen of Sciences.” To efficiently apply it in non-physical sciences, a scientist has to reject its royal robe, realise, what its limitations are, and not expect from it spectacular results in the fields, where it is able only to sweep given area of knowledge.

## Evolutionary-Neurophysiological System: Bernstein’s Brain Skyscraper

Probably the most advanced systemic description of human motor capabilities, based on evolutionary and neurophysiological data, has been invented by Nikolai Aleksandrovich Bernstein, who was inspired by earlier works of John Hughlings Jackson (Jackson, 1884; Bernstein, 1947).

Iosif Moiseevich Feigenberg, Bernstein’s friend and disciple, regarded movements as a key to understanding, how the brain works (Feigenberg, 2004, p. 44). Such a stance remains in proper accordance with this paper’s motto, expressed by James Kalat (Kalat, 2007, p. 232). Unfortunately, the keyhole for observation is very small, whereas the item to be observed—is very extensive. Thus, contemporary scientists rather remind the slaves from Plato’s cave, and not the intellectual heroes, leading triumphantly whole humankind toward a better future.

**FIGURE 1:**
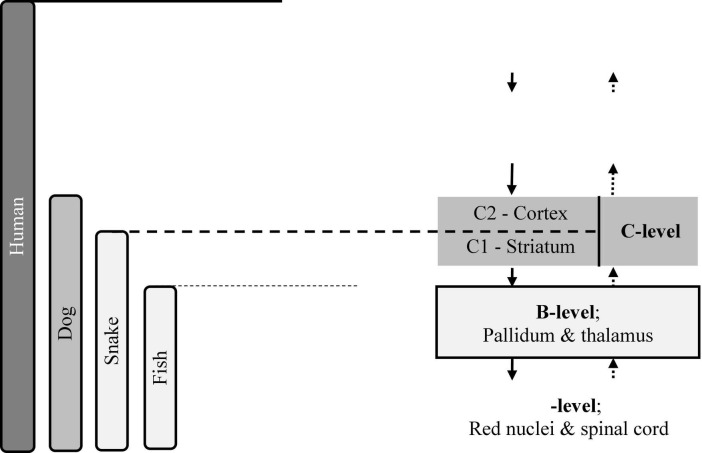
The “brain skyscraper” according to N. A. Bernstein (Petryński, 2008, p. 166; Petryński, 2016a, p. 25, modified).

Bernstein’s system—which he termed “brain skyscrapers” (Bernstein, 1991, p. 121; Bernstein, 1996, p. 99)—has been presented in Figure 1. He invented such a model and described it in his main work, “*O postroyenii dvizheniy*” (*“On the construction of movements*”) in 1947 (Bernstein, 1947), but he did not name it “*brain skyscraper.*” This term—very accurate in our opinion—appeared only in the book *“O lovkosti i yeyo rozvitii*” (“*On dexterity and its development”*), published in Russian in 1991, 25 years after Bernstein’s death (Bernstein, 1991), and in English in 1996 (Bernstein, 1996). He wrote:

“… *it is true that the human brain is a multistoried building whose stories emerged successively, one after another.”* (Bernstein, 1991, p. 121; Bernstein, 1996, p. 99; transl. Mark Latash).

However, the first full translation of Bernstein’s “opus magnum,” “On the construction of movements,” appeared in English only in 2021 (Latash, 2021).

Incidentally, the perspective presented in Figure 1 is systemic in its core, although Bernstein did not term it so, and Ludwig von Bertalanffy, considered to be the father of the theory of systems, published his seminal work only 20 years later (von Bertalanffy, 1968). Moreover, already in the 1960s, Paul MacLean developed the model of the “*triune brain*” (MacLean, 1990), similar to Bernstein’s brain skyscraper, i.e., also of systemic nature.

It seems worth noting, too, that the “intellectual skeleton” of the “On the construction of movements,” which has been published in 1947, is also cybernetic, though the seminal book of mathematician and philosopher Norbert Wiener, “*Cybernetics or control and communication in the animal and the machine,”* which marked the birth of cybernetics as a science, appeared only later, in 1948. Symptomatically, the formulation “*in an animal*” means that the origins of cybernetics are to be found—at least partly—in biology.

## Functional System: The Modalities’ Ladder

Because of the “Iron Curtain,” Bernstein’s achievements were not known in the West. The model by MacLean was regarded as “oversimplified” and “good enough for the layman.” But was this right?

Let us take a by far simpler example. There is no doubt that for a car’s movement the dynamics of fuel combustion in the cylinders are responsible. However, a good driver does not need to know its details: one needs only to know that for acceleration, one has to push the accelerator pedal. This remains in keeping—in general—with Bernstein’s Degrees of Freedom Problem (Bernstein, 1947, p.) and Andy Clark’s “*007 principles*,” which reads: “*to know only as much as you need to know to get the job done*” (Clark, 1989, p. 64).

In this context, the words of devil’s prince Woland to Immanuel Kant in the famous novel “*Master and Margarita*” sound rather ominous:

*As you will, Professor, but what you’ve thought up doesn’t hang together. It’s clever, maybe, but mighty unclear. You’ll be laughed at* (Bulgakov, 2008).

Therefore, one might say that very sophisticated theories are sometimes excellent, indeed, but they have no “cooperative power” with other theories. For example, the multidimensional theory of anxiety by Rainer Martens, Robin Vealey, and Damon Burton (Martens et al., 1990) probably reflects reality in more detail than the inverted U principle, but is in fact, too complicated to be commonly applicable. Consequently, the very applicability makes up an essential “passport to life” for each scientific theory.

Let us remember that the mind is a psychological “programming” being installed in the neurophysiological brain, which is equivalent to the “equipment.” However, the “take-home message” from neurophysiology (brain skyscraper) reads: the more complex a motor action is, the higher (and slower) the region of the brain must be engaged. However, the whole system always works like one coherent unit. Accordingly, there is no “one-to-one” assignment of given motor action (or all the more, its component) to a specific “floor” of the brain skyscraper. In the final motor operation, it is not possible to discern, what results from perception, what from attention, what from intellect, etc.

However, in motor control and psychology, just the mind comprises the main point of interest. Accordingly, let us “cleanse” the brain skyscraper from evolutionary and neurophysiological components and “distil” only the information processing ones, while preserving the same (or nearly the same) system of relations. The result may be termed the “modalities’ ladder” (Petryński, 2016a,^2019^). To avoid misunderstandings, let us term the skyscraper’s “floors”—“the levels,” and the modalities’ ladder’s layers—“the rungs.”

While assuming a functional perspective, it becomes necessary to make some small modifications. The A-level of the brain skyscraper must be divided in the modalities’ ladder into two sub-rungs: A1, responsible for maintaining posture (basic muscle tonus), and A2, which controls strength production. Further, in the modalities ladder, there is no need to divide the C-level into C1 (agility) and C2 (dexterity) sub-levels. Accordingly, the ladder contains only one C-rung. A comparison of the brain skyscraper and the modalities ladder is shown in Table 2.

**TABLE 2:** The comparison of the brain skyscraper and the modalities’ ladder (Petryński, 2016a, p. 103; Petryński, 2019, p. 48).

BRAIN SKYSCRAPER; mental-motor abilities	BASIC OPERATION; method of motor task solving	MODALITIES’ LADDER; patterns of motor operations	THEORETICAL DESCRIPTION; “physical appearance” of the movement
E-level,	No motor operation,	E-rung	
fantastic image of	POLITICS	symbolic	
reality,	(wisdom,	modality	Topology
FANTASY	anticipation)	IDEA	
D-level,	Performance,	D-rung	
accurate representation of reality, COMMON SENSE	STRATEGY effectiveness of action	verbal modality PROGRAMME	Geometry
C2-sublevel,			
net of muscle synergies,			
working organs,		C-rung	
DEXTERITY	Habit,	remote	
	TACTICS	(teleceptive)	Kinematics
C1-sublevel,	(measure-in-eye)	modality	
net of muscle synergies,		SCENARIO	
whole body,			
AGILITY			
B-level, two muscles’ synergy, MOVEMENTS’ HARMONY	Automatism, TECHNIQUE (movement smartness)	B-rung Contactceptive modality TEMPLATE	Kinetics
A-level, single muscle contraction STRENGTH, MUSCLE TONUS, (background of all backgrounds)	Reflex, STRENGTH CONTROL (feeling-in-hand) MAINTAINING POSTURE (feeling of one’s body position)	A2-subrung proprioceptive modalityCOUPLING A1-subrung kinaesthetic modality KINAESTHESIA	Dynamics Statics

If it had one more column to the left, entitled “Neurophysiological structure of brain skyscraper,” it would include neurophysiological information, which part of the central nervous system is mainly responsible for the content of the line in each of the four columns shown in Table 2. It would be consistent with the reductionist perspective, indeed, and would show, how learned the neurophysiologists are, but there is one important hitch. The system always works as one, coherent, and inseparable unit, and not as a sum of its components. Hence, its product is always an unpredictable, qualitatively, and new system effect. For example, the pallidum and thalamus play different roles in a fish, cat, and human. The division into components kills both the system and the system effects, whereas psychology and anthropokinetics, as well, deal exclusively with the system effects. A more detailed description of the brain skyscraper and the modalities’ ladder can be found in Petryński (2016a,2019).

• of motor operations—reflex, automatism, habit, performance;
• of their respective “driving mechanisms”—basic muscle tonus, strength control, technique, tactics (agility, dexterity), strategy and politics;
• of their “mental patterns”—coupling, template, scenario, programme, idea; and
• of their physical and mathematical “counterparts”—statics, dynamics, kinetics, kinematics, geometry, topology (Table 2).

In this context, one may admire the genius of Nikolai Bernstein. According to outstanding mathematician Stefan Banach, “*good mathematician sees analogies between theories, while the best of them discern analogies between analogies*” (Urbanek, 2014, p. 206). In motor control directly observable (and prone to experimental research) are only environmental stimuli and the resulting movement. Bernstein’s great predecessors, Ivan Mikhaylovich Sechenov, and his follower, Nobel Prize winner Ivan Petrovich Pavlov, identified only one mechanism of movements control: the reflex (Sechenov, 1942; Pavlov, 1973). They did not analyse the possible various modalities in motor operation construction in living beings. On the other hand, Bernstein was able to catch a glimpse of “analogies between analogies,” and discerned the deeply hidden multilevel and multimodal structure of the motor control mechanisms in humans.

In Bernstein’s theory very important is the division of the “floors,” active in each motor operation, into two groups: the main level and the background. The former works in the feedback mode (hence, it needs attention) and is responsible for what is being performed. The background works in the feedforward mode (it does not engage attention) and is responsible for how a given motor operation is being realised. Such a structure enables the efficient execution of complex operations, while making very frugal use of precious attention.

## Operational System: the Stream of Consciousness in a Motor Operation

In a motor operation, with this term, we describe any purposeful motor action aimed at solving a specific task in the environment; the visible and measurable components are, exclusively, an initial stimulus (the releaser) and the effect of muscle activity (the biological strength), i.e., physical force and/or motion. Both phenomena make up the only “keyhole,” through which we may peep on the action of the mind. Let us quote philosopher Andrzej Wohl: “*All that we dispose of*, *all that constitutes the resource of our culture*, *all the pieces of art*, *science and technology—all that results from motor activities*…” (Wohl, 1965, p. 5; transl. WP).

Let us emphasise that there are no other behaviours than motor ones because movement is the only method of manifestation of what is going on in the mind and the only method of influencing the environment by a human as well. Even if only that of the lips and tongue.

What goes on between reception of the releaser and production of movement we can only conjecture, for these phenomena and processes cannot be researched directly and experimentally. It seems worth bearing in mind that at the brink of the nineteenth and twentieth century, and in the following decades as well, a very strong impulse for the development of physics was the purely theoretical, “crazy” works of Max Planck, Albert Einstein, Niels Bohr, Peter Higgs, and many others. Respective experiments, in which the phenomena congruent with theoretical anticipation were observed, had been performed much later. For example, the apparent moonshine concepts, like the general theory of relativity, waited for such an experiment 4 years; the Higgs boson in half a century and the gravitational waves in full century. Let us add that the matter of human motor behaviour is, by far, more complex than any problem in physics. Hence, the expectation of immediate experimental verifiability of hypotheses prevents scientists from the free formulation of conjectures, being the most primeval “seeds” of science. In this respect, a highly instructive sound of words of Nobel Prize winner Niels Bohr to another Nobelist, Wolfgang Pauli: “*We are all agreed that your theory is crazy. The question, which divides us, is whether it is crazy enough to have a chance of being correct. My own feeling is that it is not crazy enough*.” It is hard to resist the impression that nowadays, the non-mathematical biology, psychology, and science on motor behaviour need just crazy ideas more than “*commonly accepted methodologies*” and computers.

By the way: the theory, being the subject of the comment of Niels Bohr, cannot count among the greatest achievements of Wolfgang Pauli and of the co-author of this concept, another Nobelist, Werner Heisenberg.

Nevertheless, self-censorship strongly inhibits the development of anthropokinetics. To publish a scientific paper (“*publish or perish*”), it must be built in agreement with the template: material—research—discussion—conclusion. Such a template promotes the “*scientists with noses in the ground*.” It is enough to adequately collect many results of experiments, to process them with what may be termed “*kitchen statistics*” (or any other “*commonly accepted methodology*”), then to name the results with the word “*conclusions*” (it is an evident, yet very popular humbug)—and so, to build one’s position in science. Unfortunately, such works do not contribute to progress in science (with the capital “S”). Nevertheless, such papers comprise the majority of the content of scientific journals and magazines. Jack Cohen and Ian Stewart wrote:

*At least 999 out of a thousand scientific papers are about complex details, but the one that we treasure and for which we award a Nobel Prize is the one that reveals a new simplicity. It is as if simplicities are all around us but scattered rather thinly. Some scientists are rather good at laying hands on them; they must have the right kind of mind, seeing the world with unusual clarity. Albert Einstein specialized in big simplicities, and so did Paul Dirac, Gregor Mendel, and Dimitri Mendeleev (Cohen and Stewart, 1994, p. 231).*

In this statement, one may identify the word “*simplicity*” with the term “*theory*,” because “*a theory is a kind of code that transforms complicated messages from nature into much simpler ones”* (Cohen and Stewart, 1994, p. 363). For, as it has been said, there is no direct experimental access to the series of phenomena and processes from the releaser to the visible motor response, let us try to build a conceptual cause-effect chain that joins both these events. While borrowing the term from William James, let us name it “stream of consciousness.” It has to be placed in the sphere of theory, i.e., in the natural environment of science. Let us emphasise that it is not possible to build such a chain while basing on “tangible” experiments. In this context rather ominously sound the words by Henry Mencken, who noticed that “*science, at the bottom, is anti-intellectual; it always distrusts pure reason and demands the production of objective fact.*”

The structure of a sensorimotor response has been described in detail by Richard Schmidt (Schmidt, 1988, p. 65). He divided it into three periods: foreperiod (FP), reaction time (RT), and motor time (MT). The RT and MT together, make up the response time (RPT).

The FP commences with the reception of a signal, i.e., a stimulus which foreruns another stimulus. The latter may initiate a motor response. Let us term it “releaser.”

The RT starts with the reception of the releaser, but there is not yet any electrical activity in the muscles; it ends when the MT starts, i.e., the movement gets observable.

The term MT denotes a period when the movement (or purposeful motionlessness, as, e.g., in targeting) becomes visible. It is over along with the termination of the movement.

In such a model, the RT is the main period, when the abstract pattern of a motor response may be shaped. The conceptual information processing cause-effect chain in a sensorimotor response—the stream of consciousness, cannot be directly observed experimentally.

The first link of the chain is stimulus **reception**. It is not “understandable” to the central nervous system but arouses sense organs. In turn, they produce neural sensory inputs, of electrical nature. They are “understandable” for the neural system and may stimulate it. This link produces awareness.

The second link is **perception**, i.e., assigning the information retrieved from memory to the specific sensory input. Thus, it creates chunks of information. This link produces consciousness.

The third link is **attention**, which, based on previous experiences, assigns specific “weight” to each chunk of information and creates their hierarchy. The most important ones make the “fuel” for further information processing. The insufficiently important are rejected and forgotten; they are not transmitted further to motivation and intellect and do not charge them.

The fourth link, **motivation**, is a “doorkeeper” to the intellect. It transmits (or not) the most important chunks of information and determines the intensity of their further processing.

The fifth link, **intellect**, makes up a specific apex in the whole chain. It is a final link of the “ascending path” (more and more abstract, less and fewer information chunks to be processed, “the preparatory sub-system”), and at the same time, the initial link of the “descending path” (less and less abstract, more and more information patterns, “the executive sub-system”). It creates an abstract pattern of the whole possible motor operation. The conceptual structure of the intellect consists of three components: intelligence, intuition, and instinct.

**Intelligence** comprises the “armed forces” of intellect and is responsible for the final assembly of a motor operation pattern. It needs the full information necessary to solve a given task.

Usually, an individual does not dispose of full information. **Intuition** is responsible for guessing the lacking information (right or wrong).

**Instinct** directs the search of information toward those regions of memory, where its discovery is most probable (Petryński, 2016a,b; Petryński, 2019).

Until this link is formed, the whole chain deals with “bare” information. Intellect produces organised, purposeful patterns, which are processed in further links.

The sixth link, **foresight**, assesses, on the base of earlier experience, the applicability of the pattern worked out by the intellect. On the descending path, it is somehow “symmetrical” to attention, which resides on the ascending path.

The seventh link, **decision**, finally transfers the motor operation pattern into execution (or not); so, it is “symmetrical” to motivation.

In the eight link, **skills**, already existing patterns (or component sub-patterns) of the motor operation are being retrieved.

The **efferent copies** make up the ninth link of the chain. They are motor operation patterns recorded in memory and enhance the execution of a similar motor operation in the future.

Finally, the tenth link in the **production of strength and movement** by the muscles, bringing about desirable effects in an environment (Table 3).

**TABLE 3:** The links of conceptual motor response information processing.

No.	Input	Link	Product
1.	Stimuli	*Reception*	Sensory inputs
2.	Sensory inputs	*Perception*	Chunks of information
3.	Chunks of information	*Attention*	Essential information
4.	Essential information	*Motivation*	Operational information
5.	Operational information	*Intellect*	Possible operation pattern
6.	Possible operation pattern	*Foresight*	Realisable operation pattern
7.	Realisable operation pattern	*Decision*	Executable operation pattern
8.	Executable operation pattern	*Skills*	Motor commands pattern
9.	Motor commands pattern	*Efferent copies*	Pattern recording
10.	Motor commands pattern	*Muscles*	Strength, movement

**FIGURE 2:**
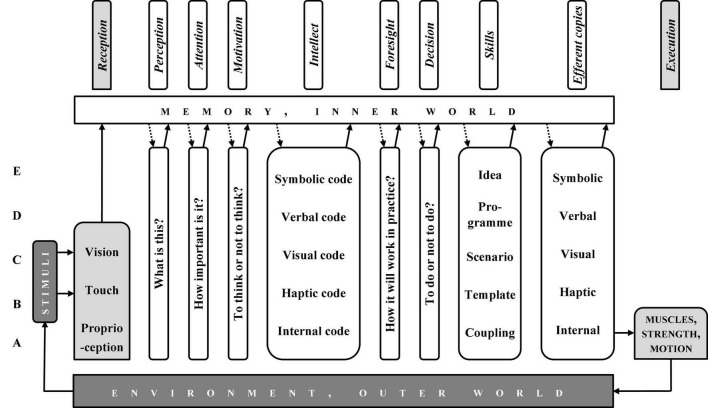
Column Diagram” of information processing in a sensorimotor response. Dark blocks, physics; light grey blocks, physiology; white blocks, psychology.

While looking at Figure 2, one may learn that from perception to intellect, the system deals with more or less “bare” information, whereas from intellect to efferent copies—with organised patterns, i.e., the systems of deliberately organised information.

Incidentally: The sensorimotor response pattern by Schmidt shows, how important the ability to anticipate is, which first appeared at the C-level of the brain skyscraper. It makes the crucial factor in the merciless, evolutionary struggle for life. It enables translation of the process of preparation of the motor response pattern ahead of the moment of the reception of the releaser. Such a process “resides” in the foreperiod (FP), which commences with the reception of a signal, which foreruns the releaser. Only the latter may launch the whole stream of consciousness resulting in a final motor operation. Thus, in the presence of a signal, the RT is reduced to near nil. Moreover, the MT may even start before the reception of the releaser, based only on anticipation.

## Joined Functional and Operational Systems: Column Diagram

The modalities’ ladder and the motor response chain are simple enough to be joined and create another system, which may be termed “Column Diagram” (CD, Figure 2). Its idea is that the motor response chain may work at each rung of the modalities’ ladder, while taking into account the specificity of information processing at a given rung. For example, the time perception “able to work,” being the base for anticipation, appeared only at C-level. Consequently, in the modalities’ ladder intuition cannot work at rungs lower than C, though a specific motor operation pattern may be prepared at C- or even D- level, and then “pushed down” for execution to B-level.

**FIGURE 3:**
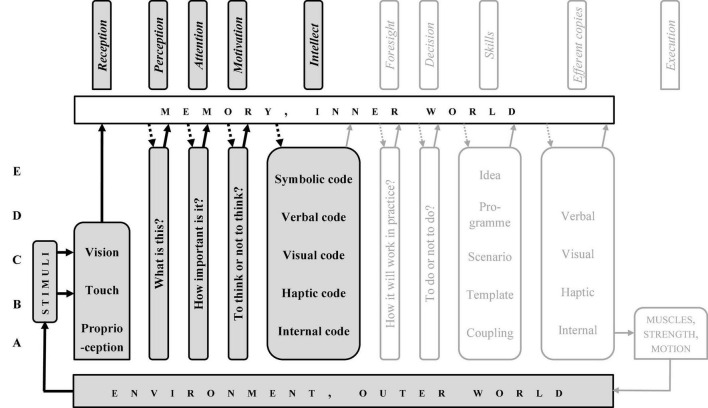
“Column diagram”; human motor operation, the preparatory path.

**FIGURE 4:**
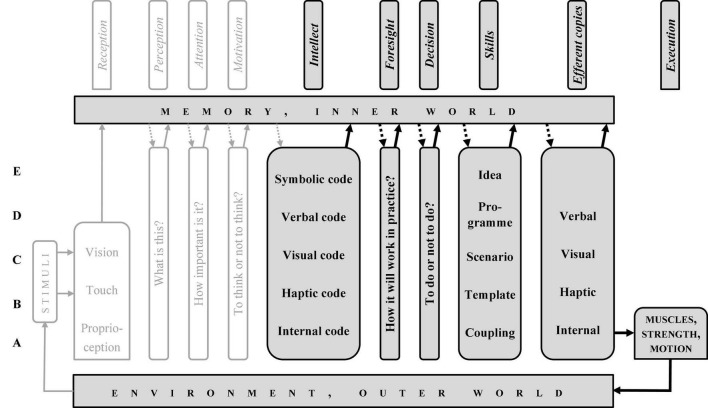
“Column diagram”; human motor operation, the executive path.

The CD, just like the stream of consciousness, may be divided into two parts: from reception to intellect (the “preparatory sub-system,” from past to present, Figure 3) and from intellect to execution (the “executive sub-system,” from present to future, Figure 4). Symptomatically, intellect, memory, and environment are components of both.

Let us look more closely at the column “Skills.”

The abstract, internal patterns result in practise with specific motor operations: reflexes at A-rung, automatisms at B-rung, habits at C-rung, and performances at D-rung. E-rung does not have its “own” motor operations.

Apropos: This issue created the main “bone of contention” between two Giants: Ivan Petrovich Pavlov and Nikolai Aleksandrovich Bernstein. According to Pavlov, each motor operation may consist of a shorter or longer chain of simple reflexes. On the other hand, Bernstein regarded that particular motor operations do not differ from one another only quantitatively (lower or higher number of reflexes), but qualitatively (various modalities of information underlying a given motor operation). Also, in by far simpler mathematics, it is not possible to solve a complex differential equation with the plain multiplication table alone.

**FIGURE 5:**
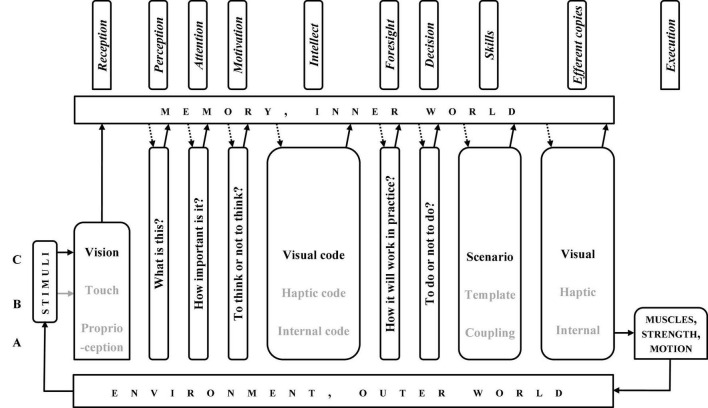
Information processing while cycling. The A- and B-rung work “in the background,” i.e., without attention focusing.

**FIGURE 6:**
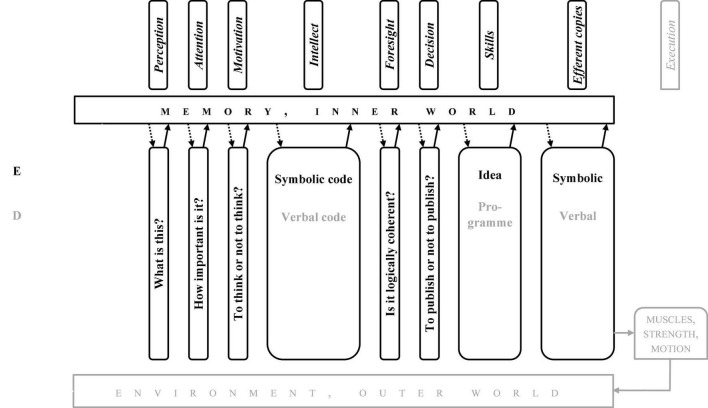
Information processing while creating an abstract mathematical construct. Even if mainly the E-rung works, the movement at A-rung is necessary to transfer the results of processing to the environment.

The CD (Figures 2–4) show all the possible chains of information processing. In fact, according to the Degrees of Freedom Reduction rule, in a specific motor operation, only the necessary rungs are active. The C-rung operation (e.g., cycling) is shown in Figure 5, and the E-rung operation (e.g., theory creation) is in Figure 6. In the latter, the motor components are reduced to near nil (they are not essential). Both these figures may be interpreted as specific illustrations of Bernstein’s “*Degrees of Freedom Reduction rule*” or Clark’s “*007 principles.*”

Incidentally, the D-rung makes a “seat” of common sense. So, while looking at Figure 6, one might discover, why an ingenious scientist, who is intensively working intellectually, can look at an egg and boil a wristwatch.

It is worth emphasising that the assumption that information processing works at all five rungs of the modalities’ ladder makes the notion of “subconsciousness” superfluous (Petryński, 2016b). For the coining of this term often Sigmund Freud is being credited. However, he wrote:

*I should also like to hear you admit that our designations—*unconscious, fore-conscious, *and* conscious *are much less likely to arouse prejudice, and are easier to justify than others that have been used or suggested—such as* sub-conscious, inter-conscious, between-conscious, *etc.* (Freud, 1920, p. 257).

In ML’s perspective, the difference between “sub-” and “fore-consciousness” is fundamental. The former concerns different modalities of the consciousness, whereas the latter concerns the various intensities of the consciousness of the same modality.

To sum up, it seems worth emphasising a very important difference between a mathematical equation and a system. The former makes a kind of universal “stiff rails” for solutions with many different sets of data. For example, the same equation may be applied to both hydraulic and electrical issues (electronic-hydraulic analogy). The same equation describes the distribution of tension in a bar in torsion and deformation of an elastic membrane under pressure (membrane analogy by Ludwig Prandtl). On the other hand, an anthropokinetic system is a disposable structure consisting of environment, body, mind, and a task to be solved with the motion. Thus, the pallidum and thalamus play different functions in the “motor creation system” not only in a fish and in a human, but also the same living being in different motor operations.

## General Discussion: Mathematics and System-Theoretical Approach

Mathematician René Thom stated that contemporary science became possible only in the 17th century, when the theory got ahead of the experiment (Sorman, 1993, p. 61). Without doubt, he thought about physics; it got then very hard, mathematical fundamentals, which enabled its eventful development and progress. Incontestable, a breakthrough was the creation of differential calculus by Isaac Newton and Gottfried Leibniz. This key moment may be regarded as the birth of “full-blooded” mathematics, as opposed to sheer calculations. It was a turning point that commenced the triumphant marsh of mathematics, not only in physics, but in whole contemporary (then) science.

However, as early as the fifteenth century, in the era of “sheer calculations,” Leonardo da Vinci remarked that “*there is no certainty in sciences where one of the mathematical sciences cannot be applied, or which are not in relation with these mathematics”* (Gauss, 2021). Nearly five centuries later, in the era of “full-blooded mathematics,” philosopher and mathematician Bertrand Russel stated that “*mathematics may be defined as the subject, in which we do not know what we are talking about, nor whether what we are saying is true.”* (Russell, 2004, p. 58). Mathematician Israel Gelfand formulated the already cited “Wigner-Gelfand principle.” Biologist Jack Cohen and mathematician Ian Stewart wrote:

*By definition, all mathematical statements are tautologies. Their conclusions are logical consequences of their hypotheses. The hypotheses already “contain” the information in the conclusions. The conclusions add nothing to what was implicitly known already. Mathematics tells you nothing new* (Cohen and Stewart, 1994, p. 234).

Philosopher and physicist, Michał Heller, remarked:

*One might assume that the simplicity of mathematical structures, with which we are modelling the world, are so different from the richness of the real structure of the world that instead of similarity we should speak rather about a resonance, which happens between the structure of the world and the structure of its mathematical models created by us* (Heller, 2011, p. 58; transl. WP).

Accordingly, mathematics sees only the aspects of reality remaining in Heller’s resonance, whereas the other ones are transparent to it. The explanation of such a phenomenon one might find in the statements of Roger Penrose and Alexander Grothendieck that mathematics is interested only in the relations between items under consideration, and not in their very nature. This is highly effective in the non-living world, where physical bodies have none of their own “personalities” and passively obey the physical laws, extrinsic to them. In this field, elegant and user-friendly mathematics makes an excellent instrument for quitely easy and precise scientific descriptions. Unfortunately, “*Physics deals with an invented, simplified world. This is how it derives its strength; this is why it works so well: Its raw material is of a type that can be placed in simple settings. Sciences like biology are less fortunate”* (Cohen and Stewart, 1994, p. 12).

As an additional comment concerning mathematics, let us quote the statement by Jack Cohen and Ian Stewart that “*a Theory of Everything would have the whole universe wrapped up; and that’s precisely what would make it useless”* (Cohen and Stewart, 1994, p. 365). Another formulation of the same in fact idea has been expressed by mathematician John Barrow, who stated that “*[*…*] paradoxically, science is only possible because some things are impossible”* (Barrow, 1999, p. vii).

In this respect, highly and instructively sound statements of Nobelist-physicist, Niels Bohr and Erwin Schrödinger. The former wrote:

*“[*…*] the existence of life must be considered as an elementary fact that cannot be explained but must be taken as a starting point in biology, in a similar way as the quantum of action, which appears as an irrational element from the point of view of classical mechanical physics, taken together with the existence of elementary particles, forms the foundation of atomic physics. The asserted impossibility of a physical or chemical explanation of the function peculiar to life would in this sense be analogous to the insufficiency of the mechanical analysis for the understanding of the stability of atoms.”* (Bohr, 1933, p. 458).

Nearly 20 years later Erwin Schrödinger wrote:

*Today, thanks to the ingenious work of biologists, mainly of geneticists, during the last thirty or 40 years, enough is known about the actual material structure of organisms and about their functioning to state that, and to tell precisely why, present-day physics and chemistry could not possibly account for what happens in space and time within a living organism* (Schrödinger, 2013, p. 4).

Incidentally, the same fact idea one might find in the famous “Faust” by Johann Wolfgang Goethe, who wrote it at the beginning of the nineteenth century:

*To know and note the living, you’ll find it.*

*Best to first dispense with the spirit:*

*Then with the pieces in your hand*, *Ah! You’ve only lost the spiritual bond.*

*“Natural treatment,” Chemistry calls it.*

*Mocks at herself and does know it* (Goethe, 2003, p. 79).

About half of the twentieth century also biologist Lucien Cuénot stated that “*in a cell, there is nothing living, but the cell itself.*” Why biology, and—even more—psychology cannot be easily “harnessed” with mathematical formalism? Probably because, unlike the physical bodies, living organisms are endowed with a kind of psychology and do not only passively obey the extrinsic physical laws, but also actively shape their relations to reality. In biology one has to do with the intrinsic purposefulness – quite “stiff”, formed in the course of evolution. On the other hand, in psychology, the intrinsic intentionality has been already developed. It is rather fugacious and shaped at a given moment by an individual. Mathematics may be useful in the description of superficial phenomena, in ordering observations. However, it is hardly useful in discovering the very nature of items under consideration in biology and psychology. Michał Heller remarked that “*the science sees the world through theories”* (Heller, 2011, p. 4; transl. WP). One might paraphrase this statement and say that “*mathematics sees the world through relations.*” However, in living beings, their “relations to peers” result to a great extent from their inner biological and psychological structure, which seems to remain, at least at the contemporary state of science development, beyond the borders of the kingdom of “Queen of Sciences.”

In such a situation the promising instrument for ordering the knowledge in both these areas seems to be the theory of systems. As opposed to a mathematical equation, the system can create a qualitatively new, unpredictable system effect. Another important difference as compared to the mathematical equation is that a system is a one-off mechanism for problem solving and to solve another similar or even the same problem, it is necessary to build a system anew. On the other hand, the mathematical equation makes it rather stiff, very convenient for scientists “rails for thinking,” which may be used many times without any alterations.

To sum up, in the area of unknown, where resides the intellectual chaos, scientists believe, not know, that it is deterministic. This conviction has been expressed by Pierre Simon de Laplace, when he created his famous “demon”; just this belief made the very fundamental of Einstein’s image of science. The unknown is being penetrated at first by philosophy, which strives to “harness” the incomprehensible world with a kind of logic. However, to become a science, this provisionally structured, yet (deterministically, hopefully) chaotic knowledge, must be properly ordered. The basic instruments for this process may be, roughly, either the “stiff” mathematics or the “elastic” system. Our thesis reads that the latter is at least not less effective than the former. Moreover, we claim that in biology, psychology, and anthropokinetics, it has a clear advantage over the “Queen of Sciences.” Therefore, we strived to present the issues of motor human behaviour from the system-theoretical perspective.

## Conclusion

The modalities’ ladder and the stream of consciousness in a motor operation are the systems somehow “orthogonal” to each other. However, they may be linked together to form what has been shown in this paper as a column diagram. The more detailed analysis of the structure built of both these systems together, along with a blueprint of a human motor operation, more detailed than a CD, termed “movements’ management matrix” (MMM), can be found in Petryński (2016a, p. 133) and Petryński (2019, p. 71).

It must be emphasised that both these systems are of non-linear nature. The links between particular elements of the stream of consciousness are non-linear; for example, attention transfers to motivation the information, which is “filtered” and selectively reinforced (or suppressed). The same concerns the rungs of the modalities’ ladder. Here the non-linearity emerges as incomplete “translatability” of information code specific to one rung into the “language” (proprioceptive, contactceptive, teleceptive, verbal, or symbolic) specific to a neighbouring rung. In this case, we have to do with a specific kind of the “epistemological obstacle,” as by Gaston Bachelard (Bachelard, 2002, p. 24). However, in this respect, such an “obstacle” has a great creative power in the abstract field of intellect. Incidentally, probably, just the non-linearity makes the main fundamental for the most important product of a system: the emergent, qualitatively new, and unpredictable system effect.

In fact, only this issue has made the main bone of contention between Pavlov and Bernstein. Great Ivan saw the simple reflex as the only mechanism “driving” any motor activity, whereas Great Nikolai discerned various modalities, non-linearly joined with each other, in different motor operations. As a result, he has built a specific “gearbox,” which is the “brain skyscraper,” thus, enabling selection of optimal modality of information processing for a given motor operation.

The system-theoretical perspective enables clear arrangement of the sciences’ underlying issues of human motor behaviour: psychology and neurophysiology (anthropokinetics), as well as physiology, anatomy, and physics (biomechanics). Together, they make the components of the more general kinesiology.

The concept of modalities’ ladder, firmly rooted in Bernstein’s theory, enables clear categorisation of motor operations, their psychological “driving mechanisms,” their internal mental patterns, as well as their physical and mathematical “counterparts.”

The practitioners RS and MS found such categorisations useful in their didactical activity. In a motor performance (D-rung), main load burdens the mental sphere, whereas the C- (habits), B- (automatisms), and A- (reflexes) rungs make merely the “armed forces” of a motor operation. The most advanced of such an operation, where the motor element prevails, is no doubt the habit. It makes a system (not a sheer sum!) of automatisms and reflexes, which in the habit should work in feedforward mode, i.e., without attention engagement. This, alone, makes the whole structure reasonable. Symptomatically, as a system, the habit always works as a coherent and inseparable unit. As such, it should be performed by a learner fluently and efficiently. However, a teacher should discern the “critical points” of the habit, which are automatisms and reflexes, and to correct just those elements, which determine the quality of a whole habit. In this respect, the crucial is identification of particular sub-operations in a habit by a teacher.

The concept of stream of consciousness joins the particular links of the cause-effect chain—reception, perception, attention etc.—in information processing during a motor operation in one coherent series. It always works as an inseparable system. Hence, in the result it is not possible to determine (or even evaluate), which part of the resulting motor operation origins in attention, which in intelligence, and which in foresight. Consequently, purely experimental, yet valuable, research of these issues, e.g., by simple calculation of the value of IQ, seems hardly possible. Moreover, a detailed analysis of these psychological mechanisms makes no sense separately at all! For example, while seen from system-theoretical perspective, memory of such gains will have meaning only when it gets included into a system consisting of environment, task, body, mind, and solution. Such a system is a one-time construct. To perform, once more, a similar or even identical motor operation, one has to build a new system. For example, if a driver has to go with his/her car from point A to point B, the elements of a system are: point A, car, driver and point B. However, when s/he goes back, we have to do with a new system: point B, car, driver, and point A. Therefore, the rule “repetitions without repetitions” makes one of the fundamentals (if not the main one) of Nikolai Bernstein’s theory. Probably, earlier foreknew this Sigmund Freud, who opposed to experimental research in psychology. As Tomasz Witkowski remarked, he thought that “*those phenomena are so elusive and delicate that it is possible to discern them only in the clinical interview, and not in an experimental research, which needs some level of standardization*” (Witkowski, 2015, p. 181; transl. WP). Let us emphasise, once more, that the systemic nature of a motor operation, along with its abstract mental pattern, seems to be hardly researchable experimentally. To perform this with any success (possible to achieve at all), a scientist must realise, what are the limitations of experimental research in this field.

Once more, it should be pointed up that mathematics, although elegant, fashionable, and user friendly, is far from being fully universal. Moreover, nowadays, the full of fantasy, smiling, and intellectually provoking Miss Mathematics, is being substituted with boring (yet reliable) Miss Calculations. The instant and zero-one sheer (if not primitive) operations in computers kill the full of fantasy and understanding mathematical analysis. In this respect, highly symptomatic is the “halting problem.” This term, roughly means that after launching a computer procedure, the scientist loses any control over it until the “number cruncher” expels the solution; solution, but not understanding. While paraphrasing mathematician Hugo Steinhaus, “*due to dissemination of computer technology, nowadays it became possible to conduct research, publish papers and achieve scientific degrees and titles while still being an illiterate”* (Steinhaus, 1980, p. 56; transl. WP). It seems worth noting that Steinhaus passed away in 1972, and the explosion of computer technology came only later.

It seems appropriate to quote another mathematician, Israel Moiseevich Gelfand, who stated that: “*Application of contemporary mathematics and physics to biology is a dead-end*… *Do not waste time on mathematics—think!”* (Latash, 2008, p. 56).

In that context, in the field of biology and psychology, the systemic approach seems to be a method of investigation far more eligible than mathematics. By now it does not produce the quantitative solutions of the issues under examination, indeed. However, mathematics (and, all the more, calculations) was not able to create a qualitative image of psychological phenomena and processes. In short, contemporary science cannot create a precise representation, like a technical drawing, of psychological phenomena. Nevertheless, scientists should strive to produce at least an impressionistic (and holistic) image of those issues, describing not their details, but their “soul.” Within this context, the system-theoretical approach seems to be promising.

The presented work is no doubts a speculative one. However, it concerns the regions of human knowledge (and science as well) accessible only by speculations. Even Richard Dawkins, known of his repartee, stated, rather timidly: “*careful inference can be more reliable than “actual observation,” however strongly our intuition protests at admitting it*.” (de Laplace, 1902, p. 15). “Rather timidly,” because some regions of science are cognisable only by “careful inference”; there is no direct experimental contact to them. In the CD directly observable are only the stimuli and the motion; all intermediate links of the stream of consciousness are accessible only by “careful inference.” The same concerns particular rungs of the modalities’ ladder. However, let us remember that in physics general theory of relativity, Higgs boson and gravitational waves must wait for “their” experiments for four, fifty, and hundred years, respectively. Moreover, the biological, psychological, and anthropokinetics issues are, by far, more complicated the physical ones. “Very symptomatic” is also the already cited statement by Michał Heller: “*science sees the world through theories.*” Concluding in this context, highly instructive readings of the aphorism by George B. Shaw: “*The reasonable man adapts himself to the world; the unreasonable man persists in trying to adapt the world to himself. Therefore, all progress depends on the unreasonable man.”*

## Author Contributions

WP, RS, and MS contributed to conception and design of the study. WP organised the database and wrote the first draft of the manuscript. RS and MS completed fragments and wrote corrections of first version. All authors contributed to manuscript revision, read, and approved the submitted version.

## Conflict of Interest

The authors declare that the research was conducted in the absence of any commercial or financial relationships that could be construed as a potential conflict of interest.

## Publisher’s Note

All claims expressed in this article are solely those of the authors and do not necessarily represent those of their affiliated organizations, or those of the publisher, the editors and the reviewers. Any product that may be evaluated in this article, or claim that may be made by its manufacturer, is not guaranteed or endorsed by the publisher.
